# Editorial: Animal Models of Stress - Current Knowledge and Potential Directions

**DOI:** 10.3389/fnbeh.2021.655214

**Published:** 2021-02-16

**Authors:** Ana Paula Pesarico, Pietro Maria Chagas, Juan Nacher

**Affiliations:** ^1^Universidade Estadual do Maranhão, Grajaú, Brazil; ^2^Faculdade da Serra Gaúcha, Caxias do Sul, Brazil; ^3^Neurobiology Unit, Program in Neurosciences and Institute of Biotechnology and Biomedicine (BIOTECMED), Universitat de València, Burjassot, Spain; ^4^Spanish National Network for Research in Mental Health (CIBERSAM), Madrid, Spain; ^5^Fundación Investigación Hospital Clínico de Valencia, Valencia, Spain

**Keywords:** stress, major depression, alternative therapy, animal models, organoselenium compounds

This Research Topic gathers different contributions that highlight novel compounds and alternative therapies in experimental models for depression, examining the best stress models for studying the effects of depressive disorders.

Excessive external stress, such as isolation stress and chronic stress are widely accepted theories in the genesis of depression (Park et al., 2019). Stress can affect the brain, as a susceptible organ, and this response can be affected by genetic influences and environmental experiences. Stress negatively affects regions of the brain which are mainly involved in the regulation of emotion including the cerebral cortex and hippocampi (Sapolsky, 2003; Holmes and Wellman, 2009).

Major depressive disorder is a leading cause of disability worldwide, affecting 4.4% of the population, making it one of the most prevalent health-related causes of human suffering (Gutierrez-Rojas et al., 2020). Furthermore, the exact pathophysiological mechanisms of depression are likely multiple, and still far from clear. Taking into account these questions about depression and stress, investigations of current knowledge and the potential directions of animal stress models in the future are necessary and important.

Finding new therapies and new antidepressant agents is of high clinical priority given that many cases of depressive disorder do not respond to conventional monoaminergic antidepressants such as selective serotonin reuptake inhibitors, tricyclic antidepressants, and monoamine oxidase inhibitors.

The first article published in this Research Topic by Bai et al. showed that electroacupuncture, a traditional Chinese alternative health care approach (Wen et al., 2020) presents antidepressant-like effects in a chronic unpredictable mild stress (CUMS) model in rats. At the preclinical level, the CUMS model is the most used paradigm in rodents, which comprises systematic and repeated exposure to varying unpredictable, and uncontrollable stressors lasting days or weeks (Isingrini et al., 2010; Bhatt et al., 2014; Pesarico et al., 2016; Shepard and Coutellier, 2018; Gall et al., 2020).

The authors investigated the specific mechanism of this therapy in depression disorder, observing that consumers and professionals know little about the underlying mechanisms of acupuncture. The authors demonstrated that electroacupuncture and fluoxetine, a second-generation antidepressant categorized as a selective serotonin reuptake inhibitor (Perez-Caballero et al., 2014), regulate the expression of key proteins in the calmodulin kinase (CAMK) signaling pathway, which are related to depression in the hippocampi of rats (Takemoto-Kimura et al., 2017; Xie et al., 2019). These results indicate that acupuncture may alleviate depressive symptoms and reduce work- and life- related burdens and stress by regulating the CAMK signaling pathway.

In a paper on “Short- and Long-Term Repeated Forced Swim Stress Induce Depressive-Like Phenotype in Mice: Effectiveness of 3-[(4-chlorophenyl)selanyl]-1-methyl-1*H*-indole,” our research group found that in the context of depression induced by stress and the antidepressant-like effect of novel molecules, 3-[(4-chlorophenyl)selanyl]-1-methyl-1*H*-indole (CMI), a synthetic organoselenium compound, is effective in abolishing the depressive-like behavior induced by repeated forced swim stress (FSS) in male mice. This animal model is a factor similar to a real-life situation (repeated and inescapable stress) (Pesarico et al.).

As alternatives to existing antidepressant treatments, many synthetic compounds have been tested, among them the organoselenium compounds, which elicit antidepressant-like effects in many animal models (Rosa et al., 2018; Birmann et al., 2019; Muller et al., 2021). Selenium is an important essential micronutrient, and selenium deficiency is associated with several disease conditions such as immune impairment. Selenium is a component of SeCys in selenoproteins, and it promotes cell cycle progression and prevents cell death (Fairweather-Tait et al., 2011; Hatfield et al., 2011). These selenoproteins include enzymes, which have important antioxidant and detoxification functions (Allan et al., 1999).

Considering the important effect of selenium in oxidative stress and the involvement of oxidative stress in depression, the authors examine oxidative stress in two brain structures, the prefrontal cortices and hippocampi, and corticosterone levels in the plasma. The secretion of corticosterone in rodents into the circulatory system is a consequence of activation of the hypothalamic-pituitary-adrenal (HPA) axis caused by stress (Holsboer, 2000; Jankord and Herman, 2008).

The results of this study demonstrate that CMI modulated the oxidative stress in the prefrontal cortices and hippocampi of mice subjected to repeated FSS. Mice subjected to repeated FSS had also an increase in the corticosterone levels and CMI regulated the levels of this glucocorticoid. The mechanism of the antidepressive-like effect of CMI in the repeated FSS model is likely its regulation of corticosterone levels and oxidative stress in the prefrontal cortices and hippocampi of mice.

The third publication in this RT is a review of the mood-related properties of acetylcholinesterase inhibitors (AChEIs), focusing on both human and rodent studies (Fitzgerald et al.). Firstly, it recalls that the cholinergic system is composed of cholinergic neurons that use the acetylcholine neurotransmitter. These neurons are activated and release acetylcholine and this system has been associated with cognitive functions, such as memory, and emotional processing (Hasselmo, 2006; Picciotto et al., 2012; Martini et al., 2018).

The authors comment in the text that many AChEIs are currently used to treat several disorders, such as Alzheimer's disease (AD) and also other dementias or glaucoma. Several studies have shown that the clinical administration of AChEIs to individuals with depression can accentuate this disease, and in some cases attenuate mania or hypomania (Mans et al., 2014; Fernandes et al., 2018).

Other studies indicate a potential antidepressant role for AChEIs in human subjects and mice (Rozzini et al., 2007; Papp et al., 2016; Fitzgerald et al., 2020).

The final study published in this topic, entitled “A Comparison of Isolation Stress and Unpredictable Chronic Mild Stress for the Establishment of Mouse Models of Depressive Disorder” by Lee et al. Regarding the fact that psychiatric disorders in humans have been linked to social stress and/or reduced social interaction, the study focus aimed to better understand the influence of different types of stress on depression in animal models. To determine the distinguishable features of two-representative animal models of stress-induced depressive disorder, the authors compared isolation stress and CUMS.

Isolation stress acts as a stressor which results in alterations in social behavior, the function of neurochemical and neuroendocrine, physiological, anatomical and behavioral changes in both animal and humans (Djordjevic et al., 2012; Yorgason et al., 2016; Castillo-Gomez et al., 2017; Filipovic et al., 2018). Acute or chronic isolation stress has been proposed to develop psychiatric and neurological disorders such as anxiety and depression, especially during the COVID-19 pandemic (Muntsant and Gimenez-Llort, 2020).

CUMS causes a number of behavioral alterations, such as loss of pleasure and apathy in a large majority of animals, considering that some animals are described as more stress-resistant. These behavioral changes, together with alterations in certain endocrine and neural variables, are similar to those found in depressive individuals. Both models cited above present face, construct, and predictive validity (Willner, 1997; Nollet et al., 2013).

The results of the last article demonstrate four-week stress, both isolation stress and CUMS induced depressive- and anxiety- like behaviors in different animal tests (open field, forced swimming, and tail suspension test). Furthermore, both isolation stress and CUMS caused alterations in the serum corticosterone levels, serotonin activity in the dorsal raphe nuclei, and microglial activity in the dentate gyrus of the hippocampi.

When the authors compared the two stress models, results indicated that social isolation strongly induced the features of depression and anxiety, as indicated by all parameters. These results indicate that isolation stress is suitable for development in animal models of depressive disorders and reveals the medical impact of social isolation in modern society, especially during the recent COVID-19 pandemic.

This RT collects together current knowledge and advances in animal models of stress. It contributes to the investigation of treatment strategies, including a selenium organic compound, AChEIs, and the treatment with electroacupuncture, in the treatment of stressed-induced depression.

## Author Contributions

All authors listed have made a substantial, direct and intellectual contribution to the work, and approved it for publication.

## Conflict of Interest

The authors declare that the research was conducted in the absence of any commercial or financial relationships that could be construed as a potential conflict of interest.
